# Autism spectrum disorder and schizophrenia: a phenomenological comparison

**DOI:** 10.3389/fpsyt.2025.1546453

**Published:** 2025-03-28

**Authors:** Tim Schnitzler, Thomas Fuchs

**Affiliations:** Phenomenological Psychopathology and Psychotherapy, Psychiatric Clinic, Heidelberg University, Heidelberg, Germany

**Keywords:** phenomenological psychopathology, self-disorder, embodied intersubjectivity, schizophrenic autism, delusion

## Abstract

A mental illness can lead to a distortion in a person’s capacity to engage with the world and other people in a variety of ways. This is particularly relevant to schizophrenia and autism spectrum disorder (ASD), which are not only historically linked, but also overlap clinically in several respects. From a phenomenological point of view, the differences or similarities between both disorders have not yet been sufficiently investigated. Schizophrenic autism can be characterized as a disorder of three interconnected dimensions, namely the self, intersubjectivity and the self’s relationship with the life-world. The present work therefore investigates differences in these three dimensions between the two disorders. One key difference is that the self-world relationship in schizophrenia can be described as unstable or fragmented, whereas in ASD it is considered stable. Finally, possible differences in the experience of delusions are discussed as a change in the self’s relationship with the world.

## 1 Schizophrenic autism and autism spectrum disorder

Being-in-the-world entails both being-for-oneself and being-with-others. A mental illness always means a restriction of the ability to encounter the world and other people in an adequate, spontaneous, and diverse way. This is especially relevant for individuals with autism spectrum disorder (ASD) or with schizophrenia. Both disorders have a strong historic connection, as Eugen Bleuler, in his seminal works, was the first to introduce both the concept of autism and that of schizophrenia (1). Taking up the term autism, Leo Kanner (2) and Hans Asperger (3) were the first to describe ASD by presenting case reports of children who exhibited challenges in social interactions that persisted since infancy, as opposed to the later manifestations observed in Bleuler’s patients. Of course, autism has undergone a significant alteration since Bleuler’s time, shifting from being a pathognomonic feature of schizophrenia to a no so logical term with the initial definition of ASD.

Eugen Bleuler defined autism as a pathognomonic feature and a basic disorder of schizophrenic psychopathology, characterized by a “detachment from reality, together with the relative and absolute predominance of the inner life” (1, p.63). Patients tend to put their personal fantasies above reality, resulting in a withdrawal from the external environment, a skewed relationship between “inner life” and “external world”, and an insufficient capacity to interact with others. It is crucial that such a withdrawal is not a voluntary decision but an existential affliction (4). While there is ongoing debate about the extent to which “withdrawal to fantasy life” is a necessary feature of schizophrenia, Bleuler’s idea of autism has provided a basis for exploring schizophrenic disturbances of selfhood and the self’s relation to reality (5).

Following Bleuler, Eugène Minkowski adopted the term “autism”, but elevated it beyond a mere description of observable phenomena by emphasizing the subject (6). He assumed that a mental state should be evaluated in connection with the subject and its environment, never in isolation (7). According to him, every psychiatric syndrome is characterized by a profound transformation in various domains. In the case of schizophrenia, this is a loss of “vital contact with reality”, which manifests itself in autism. This indicates that both “being-for-oneself” and “being-with-others” are impacted. There is a reduced attunement to the ambient world which thus affects the space of the “in-between” (8, 9). Schizophrenic autism may therefore be conceived as a disorder encompassing three interconnected dimensions: one pertaining to the self, another to intersubjectivity, and the third in relation to the life-world (10, 11).

To establish a reliable diagnosis, it is crucial to ascertain the distinguishing features of a given disorder. In contrast to classical psychopathology, which held that a diagnosis requires a typical unitary form or Gestalt (12), contemporary classification systems focus on specific symptoms in order to establish a diagnosis. This can lead to misdiagnoses, especially when differentiating between schizophrenia and ASD, due to their psychopathological similarities (13, 14). But it is one reason why interest in the overlap between ASD and prodromal symptoms of psychosis has recently grown (15). The clinical overlap complicates the distinction, as both involve no difference in social cognitive abilities such as holistic face processing (16) or theory of mind (17, 18). Furthermore, there is evidence of a shared genetic background, and epidemiological studies indicate that having a parent (19) or sibling (20) with schizophrenia increases the risk of ASD (21).

While both schizophrenia and ASD entail a modification of the self-world and self-other relationship, phenomenology allows us to identify differences in this modification in the two conditions, thereby aiding a differential diagnosis. Phenomenology, as a fundamental discipline concerned with the study of subjective experience, investigates key experiential structures such as self, corporeality and intersubjectivity. The primary objective of phenomenological inquiry is to uncover the Gestalt or essence of a disorder that is typical but individually variable and biographically anchored. Minkowski referred to this essence as the “trouble générateur” [(22), p.53], meaning the fundamental disturbance from which the various symptoms arise. In the spirit of Minkowski’s methodology, this article attempts to identify the peculiar structures underlying schizophrenia and ASD.

From a variety of scientific and clinical perspectives, attempts have been made to describe the relationship between ASD and schizophrenia (14, 23, 24). Phenomenologists have consistently maintained that a phenomenological perspective can significantly improve our understanding of ASD as well as of schizophrenia (12, 25). However, the phenomenological literature, to our knowledge, has not investigated the distinction, with the exception of an analysis of the term “autism” by (26) and a comparison of the self in both disorders using a phenomenological interview (14). Another phenomenological paper described ASD and schizophrenia as a deviation in the pre-reflective embodied relationship of self and other, without providing a comparison between the two conditions (27). The aim of this paper is to analyze the fundamental characteristics and differences between schizophrenia and ASD, using a phenomenologically informed empirical approach. Even though we do not provide any empirical facts ourselves, we refer to existing empirical studies. Using an existing literature synthesis, which is mainly based on existing phenomenological literature, our main approach is a phenomenological comparison between the disorders in the dimension of self, intersubjectivity and relation to the life-world. To our knowledge, a comprehensive phenomenological comparison between ASD and schizophrenia remains outstanding. We assume that despite a certain overlap between the two conditions in intersubjectivity, they can still be distinguished from each other in this domain. Furthermore, there are fundamental differences in the domains of the self and the relationship to the life-world.

## 2 Modification of self-constitution

Self-awareness and self-knowledge are based on several different forms of experience, which in their diversity establish different dimensions of self (for a detailed description, see 28). On the one hand, the concept of the self refers to a pole of subjective experience, a locus from which the world is perceived, while on the other hand, it also refers to a core of personality in which the fundamental characteristics of a person are stored (29). Various types of self have been delineated, dependent upon the respective conceptual frameworks applied. In this paper, we distinguish between the following three dimensions drawing on the work of Zahavi (30): (1) the minimal self, also known as ipseity (31), which is an implicit, pre-reflective self-awareness that permeates every experience without requiring explicit introspection. It includes a sense of mineness and refers to the particular manner in which something is experienced (32); (2) the interpersonal self, which develops in interbodily interactions during the first months of life and refers to the self in its immediate and unreflective engagement with others; and (3) the narrative self, which develops between the ages of 2 and 4 and refers to the ways in which individuals attempt to make sense of themselves as distinct entities and to apply narratives to develop a sense of personal coherence in their lives (33).

### 2.1 Schizophrenia

Bleuler already regarded schizophrenia as an alteration in the individual’s basic sense of self and identified impairments in patients’ ability to see themselves as the subject of experience or as the agent of an action (1). This was taken up by phenomenological psychopathology, which regards schizophrenia as a disorder of the “minimal self” (34, 35). Patients suffer primarily from a diminished “self-affection” of their experience or of ipseity (36). M. Henry understands this as the fundamental core of selfhood, a pre-reflective contact with oneself (31); its disruption leads to a diminished sense of self. This disorder is considered to be the core phenomenon of schizophrenia, and schizophrenic autism is considered to be a manifestation of the fundamental change in subjectivity (4). Other symptoms, including positive ones, are considered merely to be subsidiary (37).

Self-disturbance has been empirically documented by means of phenomenological interviews, the Examination of Anomalous Self-Experience [EASE; (38)], and has be observed in both the prodromal phase of schizophrenia as well as in early and chronic stages (39–41). Further results show that disturbance of the minimal self is characteristic of schizophrenic spectrum disorders independently of the presence of psychotic symptoms (42) and that it can predict the transition to schizophrenia in non-psychotic clinical populations and high-risk individuals, suggesting that disorder of the minimal self-precedes schizophrenia (43, 44).

As a result of this disorder, patients have to establish their own self-being, as it were, which Blankenburg regards as an essential characteristic of schizophrenic autism (11): the latter occurs where the narrative self has to fulfill the missing constitutive functions of the minimal self. Patients tend to make the normally tacit background of their experience the object of their attention and can have difficulties in perceiving the world and other people as natural and familiar (10, 45). These modifications of self-experience usually manifest well in advance of the onset of psychotic episodes (46, 47).

Of course, the description of schizophrenia as a self-disorder does not mean that patients have completely lost their subjective perspective. Rather, the disorder manifests itself in a minimal self that is fragile or constantly threatened (48). Patients may initially experience this in the form of a lack of aliveness and a sense of emptiness (9). With increasing loss of familiarity, the “mineness” of one’s movements are also diminished and must be artificially compensated for. Even the simplest actions must be consciously prepared and executed. One possible consequence of the self-disturbance and loss of implicit knowledge of the body is an increased observation of aspects of experience that are otherwise tacitly assumed and not explicitly considered (hyperreflexivity, 35, 45). However, this form of reflection is not voluntary but represents an attempt at compensation for the loss of primary self-certainty.

Pre-reflective selfhood is generally closely connected with corporeality, since the lived body is implicitly present in every feeling, perception and action, thus mediating our everyday being-in-the-world and being-with-others (49). The disruption of one’s basal self-experience therefore also manifests itself in an alienation from one’s own corporeality, a phenomenon which has been termed “disembodiment” (50, 51). The body can make itself noticeable in a disconcerting and resistant way; many of its tacit functions no longer occur spontaneously (45). In schizophrenia, an individual’s otherwise self-evident bodily embeddedness in the world is lost.

In the context of the self-disorder in schizophrenia, it is typically the minimal self that is deemed impaired, whereas the narrative self receives scant consideration (52). Nevertheless, it is reasonable to assume that in the case of a disturbance of the minimal self, the patient has difficulties coherently narrating his or her own life or episodes thereof (53). Accordingly, schizophrenia has increasingly been described in terms of a disturbance of the narrative self (54, 55), for example, in the form of impoverished and fragmented self-narratives or by patients more often referring to the present than to the past (56).

In addition to ipseity, other central characteristics for generation of a self-narrative have been identified, for example the capacity for temporal integration, engaging in reflective metacognition and autobiographical memories (54). There is evidence that all four characteristics can be affected in schizophrenia. Temporal integration and processing may be impaired in schizophrenia (57, 58). Furthermore, patients may have difficulty in modulating cognitive processes and thus directing behavior (“metacognition”, 59). Autobiographical memory has a self-identity-forming function, which consists of using past experiences to guide current or future thoughts and actions and to provide a sense of temporal continuity (60). Patients with schizophrenia can have difficulties in constructing a coherent meaning to their lives and portraying meaningful relationships between their own self and others (61–63). They also integrate the future in their narrative only to a limited extent (56, 64).

In general, self-disturbances should not be considered separately, since they influence each other. Regarding the third dimension of the self, the interpersonal self, this can be seen from the fact that the disembodiment associated with the disturbed minimal self can result in the loss not only of the bodily embeddedness in the world, but also in the social context. At the same time, narratives help to develop a normative understanding of what the individual and their fellow human beings can mutually expect from each other (54). In the case of a disturbed narrative self, this normative understanding can be impaired.

As for an analysis of the interpersonal self, this will be discussed in detail in the next section. But it should be noted here that individuals with schizophrenia may experience a disruption in their immediate social interaction with another person. For example, the ability to mimic and synchronize non-verbal behavior during interaction (65) as well as affective empathy, i.e., reacting to the emotional experience of another person, may be limited (66).

Even though a disorder of the minimal self is among the main features of the phenomenological psychopathology of schizophrenia, this approach has been criticized for overestimating the role of the self and for insufficiently considering the embedding of subjectivity in various forms of intersubjectivity (29, 67). From this latter perspective, our self is always already in interaction with other people, the subject is always intersubjective. Accordingly, the basic disturbance in schizophrenia should not be regarded as an individual phenomenon, but instead as a relational process (27).

### 2.2 Autism spectrum disorder

Even though the self may seem necessary for the conceptualization of autism (as the term’s origin in the Greek word “autos” (“self”) indicates), ASD is usually associated with difficulties or deviations in social interaction, in other words with a deviation of the interpersonal self (30, 68). Interviews using James’ (69) distinction between the self-as-object and self-as-subject revealed that autistic children were able to conceive themselves as subjects to the same degree as non-autistic children (70, 71), but were less able to conceive themselves from the perspective of another, i.e. as object. Autistic individuals are typically linked to challenges in shifting among person-centered perspectives (68). In addition, unlike non-autistic children, they would rarely refer to themselves in terms of a circle of friends or membership of a social group. These results suggest that ASD is not a global deficit in self-conceptualization but that autistic people have specific problems with their interpersonal self (68, 72). Autistic individuals have another way in their self-other relatedness and engagement including another person’s view towards themselves (68). They exhibit an apparent difficulty in engaging with the emotions of others, which are significant to both the individuals and themselves in several respects. The crucial aspect of ASD is the difficulty in understanding the meaning of social situations in a pre-reflective or implicit way (25). However, it is important to emphasize that autism can affect people to different degrees. For example, the affective response to social stimuli on the part of autistic persons may show deficits compared to non-autistic persons, but they do show some emotional reactions to other people’s moods (73, 74), indicating that the disorder of the interpersonal self is not total.

Unlike in schizophrenia, a minimal-self disorder is generally not described in diagnoses of ASD (25, 30). In the EASE, higher total and domain scores were found in schizophrenia than in ASD (14). In addition, several studies found no difference in the sense of agency on the part of autistic and non-autistic individuals (75–77), although not all of the studies agree: one found a reduced sense of agency, measured by action-effect temporal binding (78).

Regarding the assessment of self-disturbance in ASD, the heterogeneity of individuals studied must be taken into account. Most existing studies are based on autistic individuals with a reasonable level of functioning and language comprehension (70). But, as the terminology of autism as a “spectrum” already expresses, there can be great diversity in manifestations and severity. Autistic individuals can be minimally verbal (79) or even mentally impaired (80), which naturally also limits their ability to make attributions of subjectivity and of agency that are typically associated with a minimal self (81). Thus, even severely affected autistic people were ascribed a pre-reflective self-awareness, since they have perceptions, desires and emotions from a first-person perspective (25). At the same time, however, a basic self-estrangement was also observed, sometimes also an impaired propositional self-knowledge.

The narrative self refers to the way individuals construct their identities, by integrating semantic relationships between past, present, and future events. It helps individuals in comprehending their experiences, forming their self-concept, and conveying their identity to others. This requires a reflective relationship with oneself, which in turn requires interaction with another person in order to relate the perspective of the other to oneself (30, 82). Therefore, deviations in social interaction from early childhood, as observed in ASD, are likely to affect the narrative self. A study examining self-conceptualization through others’ perceptions revealed no differences between autistic adolescents and the comparison group regarding continuity and distinctiveness; however, a difference was noted in self-conceptualization of agency, potentially due to the necessity of simulation (71). When autistic individuals do talk about past experiences, they may tend to use an observer rather than a first-person perspective (83), as if they were reporting on the experiences of someone else (84).

Regarding the four prerequisites for generating a narrative self (54), there are indications that metacognitive accuracy (85, 86) and temporal integration can be reduced in ASD (87, 88), although there is no impaired ipseity. Autistic individuals may have difficulties with autobiographical memory (89, 90). When recounting their own life stories, the coherence in autistic individuals was limited (91). On the other hand, group differences disappeared in some studies when adjustments were made for the respective language skills of both groups (89, 92, 93). Autistic individuals appear to derive less personal meaning from autobiographical memories that aim to define the self-compared to non-autistic individuals, with corresponding effects on the narrative self (94). Nevertheless, the numerous autobiographical accounts of autistic people show that retrospective evaluations of one’s own self can remain mostly intact in autism [e.g. (95)]. Accordingly, recent studies have shown that autistic people have an intact *self-reference effect*, i.e. an improved ability to remember information when it is relevant to one’s own person (96, 97).

As an interim conclusion, it can be stated that in schizophrenia all three dimensions of the self can be disturbed, even if a minimal self-disorder is considered the most fundamental level of selfhood that is affected. At the same time, this view has been criticized for not sufficiently considering the intersubjective dimension of human existence itself. Which self-disorder or dimension is given priority depends on the approach, namely whether the focus is on the impact of schizophrenia on subjectivity or on intersubjectivity. A solution for this is provided by the view of schizophrenia as a relational process (27). In ASD, the minimal self is generally not considered to be impaired; instead ASD is primarily associated with a deviation of the interpersonal self, which is consistent with the diagnostic criteria, where restricted social communication and interaction are decisive. As for the narrative self, there are indeed indications that it can be impaired, but this is not considered central.

## 3 Modification of intersubjective constitution

As the body imparts the basic, often implicit understanding of other people, as well as the practical skills to interact with them, deviations in the individual’s bodily embeddedness in the world have an influence on social interaction in schizophrenia and ASD. Merleau-Ponty has emphasized that in every interaction one’s own and the other’s body are primarily perceived as interconnected in what he called intercorporeality (98, 99). In each encounter, affects are mutually exchanged and felt in bodily resonance. This results in a dynamic interplay or an interbodily resonance, whereby the other person’s expression elicits a bodily response within oneself that is in turn perceived as an expression by the other. Irrespective of potential cognitive considerations, the other person is experienced through his or her bodily expression. Nevertheless, a social encounter can also be analyzed through the lens of common sense. This can be defined as a pre-reflective understanding of the meaning of everyday situations, which is a crucial part of the intersubjective constitution of the lifeworld (100). In the subsequent section, ASD and schizophrenia are depicted as conditions that hinder both interbodily resonance and common sense.

Resources for overcoming the individualistic perspective of the self-disorder can be found in the works of Blankenburg, who describes schizophrenia as a “loss of common sense”, i.e., patients have difficulties with implicit social understanding (11). In phenomenological psychopathology, a disruption or loss of common sense has classically been associated with schizophrenia (4). Two aspects to common sense can be distinguished (29): on the one hand, knowledge of beliefs shared by a cultural group, or a set of rules governing behavior in ordinary life situations; on the other, intuitive knowledge that is characterized as a “sense” beneficial for attuning the subject and the external world. This latter side of common sense, understood as intuitive attunement, also applies to ASD. A core of the social difficulties of autistic people can be traced back to difficulties in intuitively understanding other people. Thus, in the following, both disorders will be described as a disorder of common sense (101).

Blankenburg was generally concerned with a phenomenological analysis of our anchoring in an intersubjectively constituted lifeworld and with the loss of this anchoring in schizophrenia. He described a case report of the hebephrenic patient Anne Rau, who experienced a disruption to the dialectic of the “self-evidence and non-self-evidence” of existence:

“*Every human should know how to behave and conduct himself. I did not have the requisite premises. There are so many things which are alien for me. It has simply to do with living, how to behave yourself in order not to be pushed outside, outside society. But I cannot find the right word for that which is lacking in me - that is the way I feel it. It is not knowledge; it is something that every child is equipped with. It is these very simple things a human being has the need for, to carry on life,… how to act, to be with other people, to know rules of the game*” [(102), p.59ff].

Anne Rau describes a pervasive sensation of confusion: the world no longer serves as a stable background for her experiences; everything can be subject to reflection and doubt. For her, the loss of familiarity and natural self-evidence encompasses not only herself but also other individuals and even the world itself. She is no longer aware of the “rules of the game” of social interaction, i.e. common sense.

Autistic people encounter challenges comparable to individuals with schizophrenia. Yet despite this similarity, autistic individuals cannot be said to have lost their implicit knowledge of the rules of the game of interaction. If anything, this deficiency in implicit knowledge has been present from their early childhood. Mrs A., a 27-year-old autistic student, describes how.

“*There seems to be a secret set of rules in conversations about what to say and when. Since I don’t know these rules, I just say whatever comes to my mind about everything I’m asked. Unfortunately, it doesn’t work that way. It often happens, therefore, that I am excluded step by step during a conversation when another person joins. So I mostly withdraw voluntarily if another person tries to join a conversation*”.

The two self-descriptions reveal a general perplexity and disorientation in the relationship with other people. The social world remains alien, it cannot be reconciled with the rational categories which the patients have to hand. This is why phenomenological approaches consider ASD and schizophrenia as disorders of embodied intersubjectivity (27, 29, 103). In this light, ASD and schizophrenia can be understood as a reduced participation in the intersubjectively constituted lifeworld, which leads to a reduced sense of familiarity – the prerequisite for taking the dimensions of the social world for granted. Common sense involves a pre-reflexive understanding of the meaning of everyday situations, which is only possible though the bodily-mediated practical skills of relating to others. This implicit relational knowledge rests, in turn, on tacit habits and evidence, which Straus also called “axioms of everyday life” (104). Individuals with schizophrenia and ASD can have difficulties in performing automatically the bodily habits and courses of action involved in a social interaction, instead perceiving these actions as disrupted. Everyday situations can often not be taken for granted and represent not a common background of experience but a constant challenge. This results in social isolation and alienation from the social world, even if there is no lack of will to interact [for ASD, s. (105)].

Based on the first-person reports cited above, it is clear that a disorder of common sense may occur in ASD and schizophrenia. Yet while there are similarities between the two patient self-reports, it is also possible to tease out differences. Just because individuals with both conditions perform similarly on different social cognitive tasks (17, 18) and describe difficulties understanding the rules of the game in a social context, the underlying processes for these similarities need not be identical (106).

Social cognition has been widely studied and compared in both disorders. It can be divided into emotion processing and theory of mind. While similar levels were found in both conditions for the theory of mind, there were indications of better performance in schizophrenia than in ASD for emotion processing (18, 107, 108). Nevertheless, there is a difference in the theory of mind with regard to the onset, since in schizophrenia the difficulty typically arise in adolescence, whereas in ASD, it manifest as early as the general development of this ability, around age four (109), which may explain the potentially greater impact in ASD (110).

Impairments of intuitive attunement can be empirically investigated by assigning mental states to non-verbal scenarios or by understanding indirect speech and beliefs from short stories (111). A meta-analysis found that although theory of mind (i.e. understanding other minds) is impaired in both conditions, the specific domains of theory of mind showed different profiles (112). While schizophrenia was more strongly associated with challenges in understanding verbal intentions, autistic individuals had more difficulty understanding indirect speech and *faux pas* situations, whereas inferring intentions was unaffected. Understanding a *faux pas* requires a two-step process. First, it is necessary to determine that a person has done something he should not have done, and second, it is necessary to realize that the other person has been hurt by it (113). Both steps therefore require the perception and understanding of social cues. There is some evidence that autistic individuals have more difficulty with social cues than individuals with schizophrenia. Autistic individuals show stronger impairments in their orientation to relevant social and emotional information than those with schizophrenia (114, 115). Although individuals with schizophrenia and ASD showed equally reduced visual attention to faces when emotionally evaluating complex social scenes, only autistic individuals were unable to modulate their gaze to faces depending on whether the faces contained meaningful emotional information or not (114). By contrast, patients with schizophrenia and normally developing participants looked at the faces more quickly when they contained socially relevant information. Social orientation is therefore more difficult for people with ASD. In addition, the emotional context plays a less important role in the perception of emotions by people with ASD than by those with schizophrenia (114, 115). By contrast, difficulties in emotion perception in schizophrenia are more likely to be associated with general cognitive challenges (108, 116).

During a real social interaction in which the profiles of adults with ASD, schizophrenia and a control group were compared, both patient groups were found to have poorer social skills than the control subjects. However, a more detailed analysis also revealed differences between the two patient groups (117). Even though both used language to the same extent, autistic individuals addressed fewer questions to their conversation partner than did schizophrenics. The autistic individuals were more concerned with providing information and discussing personal interests; they related the conversation to themselves rather than seeking to learn more about their conversation partner. By contrast, individuals with schizophrenia displayed a relatively high degree of interactive behavior and reciprocity. Furthermore, the schizophrenia group – and this result is somewhat surprising, contradicting other studies (114, 115) – was characterized by poorer non-verbal behavior (in the form of gaze and voice modulation and affective reactions).

An intuitive attunement that occurs within the framework of common sense is grounded in corporeality and emotional experience. It implies that two interacting participants attune themselves bodily and emotionally to each other in an inter-corporeal resonance (118). Analogous to common sense, schizophrenia (119, 120) and ASD (119, 120) were associated with a reduced interbodily resonance. On the one hand, the reduced embodied emotions and intentions make it difficult for other people to resonate with an individual who has schizophrenia or ASD (121–123). On the other hand, in both conditions, the expressive qualities of other people can only be understood to a limited extent, partly due to difficulties with holistic perception (16). However, autistic people’s difficulties in recognizing emotions are even more pronounced than in people with schizophrenia (108). Patients with ASD and schizophrenia show only limited ability to directly grasp emotions in an empathic act and must instead cognitively integrate individual perceptual elements into an overall meaning. This can complicate affective engagement and some patients with ASD or schizophrenia require explicit instruction in order to assign emotions to facial expressions. A weak interbodily resonance between individuals during an interaction reduces the openness of the participants and thus further restricts social interaction (118).

Although both conditions result in challenges with common sense, ASD is more distinctly marked than schizophrenia by a reduced intuitive understanding of social cues, such as facial expressions. Autistic individuals can experience social reality as distant, opaque, or challenging to grasp intuitively. In schizophrenia, intersubjectivity is less impaired by diminished intuitive social understanding; instead, transitivism can arise, wherein the patient’s states are projected onto another individual (27). From a phenomenological point of view, the differentiation between one’s own self and the other is automatically constituted in every experience as an aspect of non-reflective self-awareness (48). In case of a disorder of the minimal self, the schizophrenic patient may struggle to maintain self-awareness while adopting another person’s perspective.

The difficulties in understanding the meanings and contexts of the intersubjectively constituted world can lead to the adoption of an observer perspective in ASD and schizophrenia. At the same time, there is increased self-observation and self-evaluation, a phenomenon that can be succinctly described as hyperreflexivity. Although this term is generally associated with schizophrenia, it also appears relevant to ASD in social contexts, if hyperreflexivity is interpreted as a compensatory mechanism for reduced familiarity and common sense (124). However, whereas in ASD this pertains to misunderstood social norms, and autistic individuals frequently attempt to conform to more socially acceptable conduct, in schizophrenia hyperreflexivity can affect the entire self. Due to the disruption of basal self-awareness in schizophrenia, every bodily action can become alienated and things that were once taken for granted and familiar suddenly become dubious and strange.

Autistic persons with high levels of functioning may seek techniques to decipher or disentangle the intentions or thoughts of others. By observing others, they develop an explicit system of rules that can enhance the functionality of their interactions to some degree (27). In schizophrenia, by contrast, such compensatory techniques are uncommon. One reason could be that social understanding in general and the implicit understanding of social cues in particular appear to be more severely reduced in ASD than in schizophrenia. Nevertheless, ASD patients’ difficulties in establishing a familiar relationship and understanding the other person cannot be fully compensated for in this way, as it is not a matter of *knowing-that*, but of *knowing-how*. Consequently, autistic individuals are often unable to definitively determine which rule should be applied in a given circumstance.

## 4 Modification of relation to the life-world

In this section, the differences between ASD and schizophrenia in terms of the individual’s experience of the life-world, specifically focusing on the phenomena of space and time, will be analyzed. The life-world, according to Edmund Husserl (125), refers to the world as it manifests in natural experience, perceived as inherently given, and in contrast to the scientific concept of the world.

### 4.1 Schizophrenia

Analogous to the EASE (see Section 1, above), researchers have undertaken an “Examination of Anomalous World Experience” (EAWE) (126) to investigate disturbances in schizophrenia regarding the lived world, including the domains of space, time, and other persons. Disruptions within these domains have long been acknowledged as fundamental characteristics of schizophrenia and could be linked to disturbances in self-experience (127). According to this view, self-disorder in schizophrenia is closely linked to a change in the perception of external stimuli. In particular, fragmentation in the experience of space and time has been identified as a core feature of schizophrenia, (128): Space can be experienced as a collection of unrelated objects. In contrast, time can be fractured, resulting in individual moments being perceived as disconnected and thus as extraneous. Lived space refers to a bodily subject’s surroundings and sphere of action that the subject pre-reflectively “inhabits”. The alteration of lived space in schizophrenia can manifest itself in three related ways. The world no longer appears as a stable background of our experience and may be perceived by patients not as natural or familiar, but as confusing (12), which is due to various perceptual disorders:

First, it appears that patients can lose the sense of a subjective center (129, 130). The background of their environment can come to the fore, so that it is the space between the objects rather than the object themselves that stands out. These experiences can be perceived as threatening. Another characteristic is that lived space is no longer perceived as a whole but as a series of individual fragments. Thirdly, the objects themselves can be perceived as altered, as in the experiences of micropsia, macropsia, and dysmegalopsia (131). It is possible that they are perceived as fragmented or inconsistent in terms of shape or size [e.g.: “I have to put things together in my head. If I look at my watch I see the watch, watchstrap, face” (132)]. Although analogous challenges arise in ASD, they manifest solely within social settings, mostly due to the inability to perceive specific facial features holistically. But in schizophrenia, this fragmentation may encompass entire scenes (133). Furthermore, lived space is often no longer perceived as a space of possibility but rather as limited or deformed. In schizophrenia the world thus generally loses its familiar coherence. Even if patients know what they are supposed to do, they sometimes no longer know how to do it and they have to concentrate on every single movement to fulfill their intentions (134). What can go wrong is not one’s cognitive knowledge but one’s bodily-mediated embedding in a self-evident context of meaning, or what Merleau-Ponty called “operative intentionality” (49). By this Merleau-Ponty meant a practical directedness toward the world without explicit thematization, manifesting itself in a seamless capacity to use objects and to navigate in social space. It creates a background texture within the field of experience, thus forming our basic connection to the world, on which the objectifying intentionality is founded.

The phenomenological problem of temporalization in patients with schizophrenia concerns not the subjective experience of time, which is usually intact, but the very temporal constitution of existence. Disorders of temporality have been attributed to a fundamental fragmentation of the “intentional arc” (135, 136). Normally, the intentional arc, enabled through passive syntheses, establishes a seamless connection between past, present, and future, thus generating temporal continuity (49). The connection is based on the retentional-protentional structure of time-consciousness (137). Retention refers to the ability of consciousness to hold onto not only current perceptions but also those of the immediately preceding moment, while protention refers to the anticipation of an immediately forthcoming experience. In schizophrenia, this temporal synthesis is only possible to a limited extent, resulting in micro-gaps of conscious experience and making a coherent experience over time no longer possible. This affects the individual’s entire perception, thinking and action. Retentional processes still enable the appropriation of previous experience, although with a temporal delay (136). If protention fails, the disactualization of inappropriate associations will likewise fail. This allows disruptive thoughts to penetrate the gaps in the intentional arc, causing the subject to be surprised by themselves. The patient may therefore be limited in his or her ability to focus on the future, remaining absorbed by what is currently appearing in his or her consciousness.

The minimal self-disorder and the fragmentation of the intentional arc can result in a profound disturbance of the familiarity of the past, which Blankenburg describes in more detail as a “lack of continuity backwards” (102). Patients can find it impossible to relate a current experience to what has happened before, whether yesterday or in childhood. Their own existence lacks an origin, with the result that moving forward is equally denied. Every action requires an extra start – a start which healthy persons have always already taken. Heidegger refers to this as the “*a priori* perfect”, in which the biographical development of individual human Dasein is sedimented (138). For patients with schizophrenia, the certainty that life will take its usual course despite changing circumstances is reduced, and everything that should have an *a priori* character can, in certain respects, take on an *a posteriori* character.

### 4.2 Autism spectrum disorder

In schizophrenia, alterations in spatial and object perception, together with temporal disruption, are linked to a transformation of the entire perceptual field. The fundamental being in the world is altered. In autism, by contrast, the individual’s embeddedness in the world is not fundamentally impaired and so the relationship with the world has no disorder of operative intentionality. Generally, lived space is not experienced as fragmented and fluctuating but as stable. However, autistic individuals frequently acquire a unique sensory and perceptual manner of experiencing at a young age (139), a trait which bears similarities with the micro- or macropsia sometimes experienced by schizophrenics. Due to a different embodiment, autistic people may experience a sense of being addressed by other things in their environment, a phenomenon which can manifest, for instance, in an increased fixation on non-social objects (140, 141), and which is not typically observed in schizophrenia. Sensory hyper- or hyposensitivity in autistic individuals can manifest in every sensory organ (142) but is constant and is experienced more as confusing than threatening. Sensorimotor characteristics result in a distinct manner in which autistic individuals make sense of their world (143), which, does not disintegrate into individual parts, but continues to appear as familiar and as a stable background of our experience. It is not the world itself that appears confusing, but rather the social sphere (27).

ASD has also been associated with atypical time processing (144), but the inner time experience of autistic individuals is not essentially disrupted (145). Phenomenological interviews on the experience of time in ASD have revealed that individuals exhibited a “factual experience” with respect to time (146): the present was mostly described in terms of isolated facts that were interpreted as an extension of the past, and the future was described stereotypically or as shaped by routines. As long as autistic individuals can hold on to their performance of pleasurable activities, such as special interests or the fixed daily routines they use to structure their subjective experience of time (147, 148), they seem to experience time comparably to non-autistic individuals and the present can pass uninterrupted (145). By implementing stereotypical behavior, they can ensure that the present continues unchanged and the uncertainty of the future is diminished, which, however, comes at the cost of a certain foreclosing of future possibilities [(149), p. 106]. When external changes arise, autistic individuals can be interrupted in their continuous experience of time, but the retentional-protentional structure of time-consciousness is not affected (145). They can therefore continue to integrate past events into their current experience and also focus on the future, even if this may be associated with fear and uncertainty, in contrast to the severe impairment, irrespective of the subject’s current activity, which occurs in schizophrenia (150).

### 4.3 Delusion as a modification of the relationship with the world

Delusional experiences lead to a fundamental change in the sense of belonging to the world and thus to being itself. Even if delusion is to be examined here as a disturbed relationship with the world, the analysis also shows how the three altered dimensions presented here are interconnected, since delusion also leads to a kind of alienation from the social world and, at least in the case of schizophrenia, to a profound self-world transformation (37, 151). While schizophrenia is typically associated with delusional experiences and this indeed constitutes a diagnostic criterion (152), the incidence of psychosis in ASD is also higher than in the non-autistic population [23.50% vs 0.91% in a longitudinal register-based study; (153)].

Jaspers characterizes delusions as the creation of a new, idiosyncratic sense of reality (154). Reality is not predefined; it is created through ongoing interactions with the environment and social exchanges involving reciprocal perspective-taking (155). For reality to transcend mere subjectivity, engagement with the external world requires an objectivity of perception. This objectivity of perception is achieved by considering several viewpoints, which occurs in two ways: first, sensorimotor engagement with the environment provides an individual with a multiplicity of perspectives that transcend and relativize the current one; second, objects are seen to be perceptible not only for oneself but also for others, perhaps from other perspectives, whereby perception gains its real objectivity (156). Difficulties in perspective-taking have been described in both ASD and schizophrenia (157), which may be a reason why both conditions predispose people to delusions.

In schizophrenic delusion, patients perceive their surroundings in a distorted and unreal way; things lose their familiar meanings (131). The intentionality of perception is reversed, as it were: patients are no longer directed at their surroundings and other people but instead things and people appear to be directed at them. They become the center of every gaze and every significance (155). This is then sometimes delusionally attributed to the intentions and actions of an external entity. This subjectivization of perception undermines the fundamental confidence that was previously placed in the intersubjectively constituted world (158). What hitherto seemed unreal or artificial is now interpreted to justify the altered perception: it has been created intentionally by foreign agents, thereby establishing a new delusional (pseudo-) objectivity. The delusion’s exonerating effect stems from the rationale it offers in accounting for the transcendental perceptual disturbance—for instance, by ascribing it to persecution by outside forces. An intersubjective discourse concerning a world that appears incomprehensible is abandoned in favor of a delusional sense-making that is reinforced through delusional work into a “meaningful” structure. Although patients retain the ability to envision the thoughts of others, their theory of mind is not fundamentally impaired; rather, it is modified to the extent that the patients relate every conceivable perspective to themselves.

Delusional experiences in schizophrenia have been associated with a disorder of the minimal self (33) and the intersubjective constitution of the sense of a Self (159). The sense of ownership is a crucial aspect of the minimal self, indicating that it is I who performs an action. Phenomenological investigations have shown how schizophrenic patients make misattributions about the agency of bodily movements (33, 160). But the intersubjective side of the self in the form of schizophrenic autism has also been associated with delusion (159). So, difficulties in the expressive-perceptual attunement between the subject and the world as well as fragile intersubjective connections have been pointed to as fundamental for the understanding of the development of delusions.

An increasing number of studies report a higher prevalence of delusions in ASD (153, 161) than in the non-autistic population, although the manifestation of symptoms in ASD and schizophrenia show differences in the characteristics of paranoid ideation (162). As a common trigger for the onset of delusions in ASD, psychosocial stress factors have been identified (163, 164). Since stress also plays a crucial role in the development of delusion in schizophrenia (165) and comparisons between both conditions regarding this relationship are currently lacking, the role played by stress does not allow for a distinction between the two disorders. Another possible influencing factor in delusion (166, 167), which is described in both disorders, is a disturbance of theory of mind (168).

Other underlying factors have been identified (162). While individuals with schizophrenia themselves indicate that perceived victimization and threat were relevant to the onset of delusions, those with ASD emphasized social cynicism, representing a negative evaluation of human nature and social interaction. Beside differences in the influence of social interaction, the appearance of delusion in schizophrenia has been linked to distortions in the way perceptions are attributed (166, 167); however, this was not the case in ASD (169, 170). Blackshaw et al. (169) suggest that the experience of delusions in schizophrenia is associated with an externalizing attribution bias, which acts as a protection against contradictions in perception. Externalizing bias refers to the tendency of people to attribute the causes of negative events to external factors, which thus relates to the sense of agency and, consequently, to the minimal self (81). Here, there is an overlap between empirical and phenomenological-theoretical research, as both associate the occurrence of delusional experiences in schizophrenia with the minimal self. Since this is considered to be disturbed in schizophrenia but not in ASD, this could be taken to indicate how delusional experiences differ between the two disorders and why they occur much more frequently in schizophrenia. On the other hand, delusional experiences in ASD have been more closely associated with social impairments, which lead to autists not understanding social interactions (162, 169).

The difficulties autistic people face in terms of common sense result in reciprocal challenges during social interactions, which appears to be more pronounced in ASD than in schizophrenia. This complicates mutual understanding and taking each other’s perspectives, resulting in the inability to consistently rectify misunderstandings. Difficulties in implicit mutual understanding and perspective-taking may in turn contribute to delusions in autistic individuals. In contrast to schizophrenia, however, there is no permanent subjectivization of perception; autistic individuals’ delusional experiences are typically brief and do not meet the criteria of duration typifying schizophrenia (171).

The different limitations to intersubjectivity in ASD and schizophrenia, along with the subjectivization of perception, the occurrence of a disturbed minimal self and externalizing attribution bias in schizophrenia, may elucidate the differences in delusional experiences between both conditions. This may indicate that the delusional experiences of autistic individuals are generally transient and do not have the duration required to count as schizophrenia (152, 171). Following Jaspers, it is likely that these delusional experiences in ASD are not a true (schizophrenic) delusion, but only paranoid delusions, arising from a distorted view of social contexts rather than from psychosis.

## 5 Summary and conclusion

Schizophrenia and ASD are not only historically linked by the notion of autism but also overlap in clinical, epidemiological and genetic terms. Nevertheless, from a phenomenological point of view, the two can be differentiated (for an overview see **Table 1**). This paper has sought to ground such a differentiation by considering three dimensions, namely disorders of (1) the self, (2) of intersubjectivity, including interbodily resonance and common sense and (3) of the relationship with the life-world.

1. At the level of self-constitution, schizophrenia is considered a disorder of the minimal self, since the implicit, pre-reflective self-awareness that permeates all experiences is often reduced. This is closely connected with corporeality, resulting in disembodiment. The body might manifest itself in a disconcerting and resistant manner; numerous of its implicit functions can no longer be spontaneously realized. In addition to a disorder of the minimal self, however, schizophrenia should always be considered in relation to the effects on intersubjectivity, as both forms of self are interrelated and schizophrenia can also be considered a disorder of the interpersonal self. By contrast, ASD displays no such fundamental change in the minimal self. Instead, a disorder of the interpersonal self is decisive.
  As far as the narrative self is concerned, evidence has been found that it can be impaired in both schizophrenia and ASD. Changes in the narrative self have received increasing attention in recent years, at least in cases of schizophrenia, although these changes are not considered fundamental. Even if the differences in the narrative self in both disorders have yet to be compared in detail, they can be assumed, as the minimal self is key to generating a narrative self, but a disruption of the minimal self only occurs in schizophrenia.
2. Both disorders can be seen to exhibit distinct characteristics in terms of challenges to social interaction, above all in reduced interbodily resonance, as well as restricted engagement with or disruption of embodied common sense. Reduced embodied resonance arises because, on the one hand, the expressive qualities of the interaction partner are only partly understood and, on the other hand, one’s own emotions are expressed differently or in attenuated form. This leads to mutual difficulties in interaction and the other person is perceived as unfamiliar and mysterious. The incorporation and execution of norms and strategies related to behavioral flexibility are essential elements of the capacity to deal with social difficulties. ASD and schizophrenia can thus both be regarded as a reduced common sense, as a limitation of our pre-reflexive understanding of the meaning of everyday situations. A lack of tacit knowledge about social situations in turn limits the ability to take the perspective of others.
  One difference between the two disorders in relation to common sense could be that, autistic individuals have greater difficulties in the implicit understanding of social situations than individuals with schizophrenia. Accordingly, it is only autistic individuals who try to compensate for social difficulties by developing a set of explicit rules. However, this explicit knowledge is unable to substitute for the implicit interactive knowing-how that relies on intercorporeality.
3. A fundamental characteristic of schizophrenia can be identified in the fragmentation of the experience of space and time, while the experience in ASD can generally be regarded as stable. In schizophrenia, the experience of lived space is altered, as patients can lose the sense of a subjective center and space is no longer perceived as a whole but rather as a series of individual items. In contrast, these changes do not appear in ASD. However, the perception of individual objects can be altered in both disorders. These changes can lead, in schizophrenia, to lived space being perceived as threatening, whereas in ASD it tends to be perceived merely as confusing.
  Schizophrenia can furthermore involve impaired operational intentionality, which manifests as a diminished practical orientation towards the world and renders objects unusable without explicit thematization. Conversely, this intentionality is not substantially compromised in ASD. Nevertheless, the ways in which autistic embodiment deviates may also impact one’s interaction with the world.
  The retentional-protentional structure of time-consciousness, which normally enables temporal continuity, may be impaired in schizophrenia. This can lead to micro-gaps in consciousness, a fragmentation of thought, as well as a limited ability to focus on the future. This structure is not affected in ASD. In the latter, time experience can instead be altered in such a way that individuals try to make their lives uniform and predictable, for example, by structuring it through stereotypical behavior. When external changes occur, patients can be interrupted in these activities, which can then cause anxiety; however, the experience of time is not fundamentally disrupted as in schizophrenia.
  In both conditions, the perspective of the other can only be taken to a limited extent, potentially contributing to the emergence of delusional tendencies. In schizophrenia, however, there is a subjectivization of perception, which is why intentionality is reversed and the patient no longer relates to his environment, but the objects relate to him. This is ultimately experienced as a result of action by some external agency and a delusional experience occurs. In addition, schizophrenia can involve a disorder of the minimal self or an externalizing attribution bias, which is also associated with the occurrence of delusions. Neither of these occurs in ASD, which may be one reason why delusions on the part of this group are of brief duration. Instead, social misunderstandings seem to have a stronger influence on the development of ASD patients’ delusions.

**Table 1:** Overview of the most important similarities and differences between schizophrenia and ASD, differentiated according to the three interconnected dimensions of self, intersubjectivity and relation to the life-world.

	Schizophrenia	Autism Spectrum Disorder
Self-Constitution	- disorder of the minimal self/impaired basic self-affection	- intact basic self-affection
	- interpersonal self is reduced - deviations of the interpersonal self are fundamental - there are indications of a narrative self-disorder, but they are not considered central	
Intersubjectivity	Both can be characterized as: 1. a reduced participation in the intersubjectively constituted lifeworld/common sense 2. a diminished interbodily resonance Social understanding in general and the implicit understanding of social cues in particular appear to be more severely reduced in ASD than in schizophrenia.	
Relation to the life-world: to lived space: to time: delusional experience:	1. patients can lose the sense of a subjective center 2. impairment of bodily/operative intentionality 3. no preference on non-social objects ⇒ relationship with the life-world is fragmented	1. Patients keep their subjective center 2. no impairment of operative intentionality 3. increased fixation on non-social objects ⇒ relationship with the life-world is stable
	- objects themselves can be perceived as altered e.g.: micropsia, dysmegalopsia e.g.: sensory hyper- or hyposensitivity	
	- fragmentation of the intentional arc/disturbed retentional-protentional structure - no stereotypes	- intact retentional-protentional structure - implementation of stereotypes so that the present continues unchanged; the flow of time can be interrupted by external changes
	- difficulties in perspective-taking	
	- subjectivized perception or “self-centrality” - externalizing attribution bias/minimal self-disorder as influencing factors for delusion ⇒ resulting in persistent delusional experience	- no subjectivized perception - no externalizing attribution bias/minimal self-disorder ⇒ resulting in short-lasting delusional experience

As the present paper has sought to demonstrate, there are certain similarities between ASD and schizophrenia in terms of three fundamental dimensions of experience; however, there are also crucial distinctions. Schizophrenia is a disorder that affects the perception of self and world at a more fundamental level than does ASD. The present work therefore has a practical application, since a deeper understanding of how ASD and schizophrenia differ psychopathologically in their basic structure or gestalt can help to distinguish between them in difficult cases of differential diagnosis. Enhanced psychopathological understanding may be especially pertinent in cases where both disorders require differential diagnosis and differentiation becomes challenging due to the absence of early developmental history of the patient, necessitating decisions based solely on the present psychopathology. In such cases, it could be more auspicious, not to overly emphasize intersubjectivity, as tempting with ASD; instead, it is essential to investigate the individual’s self-experience, spatial perception, and temporal experience more thoroughly. When an individual reports experiencing delusions, it is crucial to explore their duration, as these phenomena are mostly linked to schizophrenia but may also manifest in ASD.

A limitation of our paper arises from the phenomenological method, which is based on fundamental structures such as self, corporeality and intersubjectivity as well as clinical experience. However, no separate empirical investigation was carried out to empirically verify our assumptions.

## Data Availability

The original contributions presented in the study are included in the article/supplementary material. Further inquiries can be directed to the corresponding author.
